# Quantification and Radiological Risk Estimation Due to the Presence of Natural Radionuclides in Maiganga Coal, Nigeria

**DOI:** 10.1371/journal.pone.0158100

**Published:** 2016-06-27

**Authors:** Matthew Tikpangi Kolo, Mayeen Uddin Khandaker, Yusoff Mohd Amin, Wan Hasiah Binti Abdullah

**Affiliations:** 1 Department of Physics, University of Malaya, 50603, Kuala Lumpur, Malaysia; 2 Department of Geology, University of Malaya, 50603, Kuala Lumpur, Malaysia; 3 Department of Physics, Federal University of Technology, Minna, Niger state, Nigeria; ENEA, ITALY

## Abstract

Following the increasing demand of coal for power generation, activity concentrations of primordial radionuclides were determined in Nigerian coal using the gamma spectrometric technique with the aim of evaluating the radiological implications of coal utilization and exploitation in the country. Mean activity concentrations of ^226^Ra, ^232^Th, and ^40^K were 8.18±0.3, 6.97±0.3, and 27.38±0.8 Bq kg^-1^, respectively. These values were compared with those of similar studies reported in literature. The mean estimated radium equivalent activity was 20.26 Bq kg^-1^ with corresponding average external hazard index of 0.05. Internal hazard index and representative gamma index recorded mean values of 0.08 and 0.14, respectively. These values were lower than their respective precautionary limits set by UNSCEAR. Average excess lifetime cancer risk was calculated to be 0.04×10^−3^, which was insignificant compared with 0.05 prescribed by ICRP for low level radiation. Pearson correlation matrix showed significant positive relationship between ^226^Ra and ^232^Th, and with other estimated hazard parameters. Cumulative mean occupational dose received by coal workers via the three exposure routes was 7.69 ×10^−3^ mSv y^-1^, with inhalation pathway accounting for about 98%. All radiological hazard indices evaluated showed values within limits of safety. There is, therefore, no likelihood of any immediate radiological health hazards to coal workers, final users, and the environment from the exploitation and utilization of Maiganga coal.

## Introduction

The ever growing challenge of population explosion, human civilization, rapid urbanization, and high level industrialization has led to increasing demand for energy and power generation all over the world [1–3]. Whereas many nations are developing their nuclear energy base and others expanding their biomass and wind energy capacities, coal has proven to be the most abundant, most versatile, readily available, and easily assessable source of fossil fuel [4, 5]. Previous studies have highlighted significant contributions of coal to the sustenance of rapidly expanding social, economic, energy, and industrial sectors of many developed and developing nations [6–16]. However, the environmental impacts and human health challenges associated with coal exploitation and utilization demands urgent attention.

Coal is a sedimentary rock whose organic and inorganic mineral aggregates contain varied concentrations of naturally occurring radioactive materials (NORM) including uranium (^238^U, ^235^U) and thorium (^232^Th) decay chains as well as radioactive potassium (^40^K) [17–19]. Concentrations of these primordial radionuclides, though dependant on the geological formations of coal, are comparable to the average radioactivity of the earth crust [6, 15, 20–24]. Mining, processing, and combustion of coal redistribute and concentrate the radionuclides in the environment, thereby enhancing environmental radiation levels above normal background [24]. This results in higher dose delivery not only to coal workers but also to final users and the general environment [5, 25–27]. Black-lung disease is prevalent among coal miners due to inhalation of coal dust in quantities that are beyond the cleaning mechanism of the lungs [14,28]. Increased cancer risk due to external gamma ray exposure to coal has also been reported among coal workers and the population living close to minery [14, 26, 28–29]. It is therefore necessary to evaluate the radioactivity levels of coal in order to assess the radiological impacts that may be associated with its exploitation and to develop functional plan and radiation dose control framework for coal workers and the general public.

Extensive research have been carried out to assess the radionuclide contents of coal deposits around the world [1, 2, 5, 13, 17–19, 26, 30–32], but information on the radioactivity of Nigerian coal is relatively limited. Yet Nigeria is endowed with abundant coal deposits with enough capacity to generate up to 30% of its energy needs. No data has been reported on the radioactivity of Maiganga coal which is presently one of the main investment targets by foreign investors and the Nigerian government for power generation. The objective of this study therefore, is to quantify the natural radioactivity levels of Maiganga coal, Northeast Nigeria, and to estimate the radiation hazard indices from the activity concentrations of ^226^Ra, ^232^Th, and ^40^K. This will help in predicting any radiological hazard to coal workers, final users, and the general public from its exploitation. The results of this study will provide comprehensive baseline radioactivity data that will assist the government in developing effective coal power plant project and radiological safety management of the project. The findings of this investigation will also be relevant for coal investors to determine the quality of Nigerian coal.

## Materials and Methods

### Sampling site

**Fig 1:**
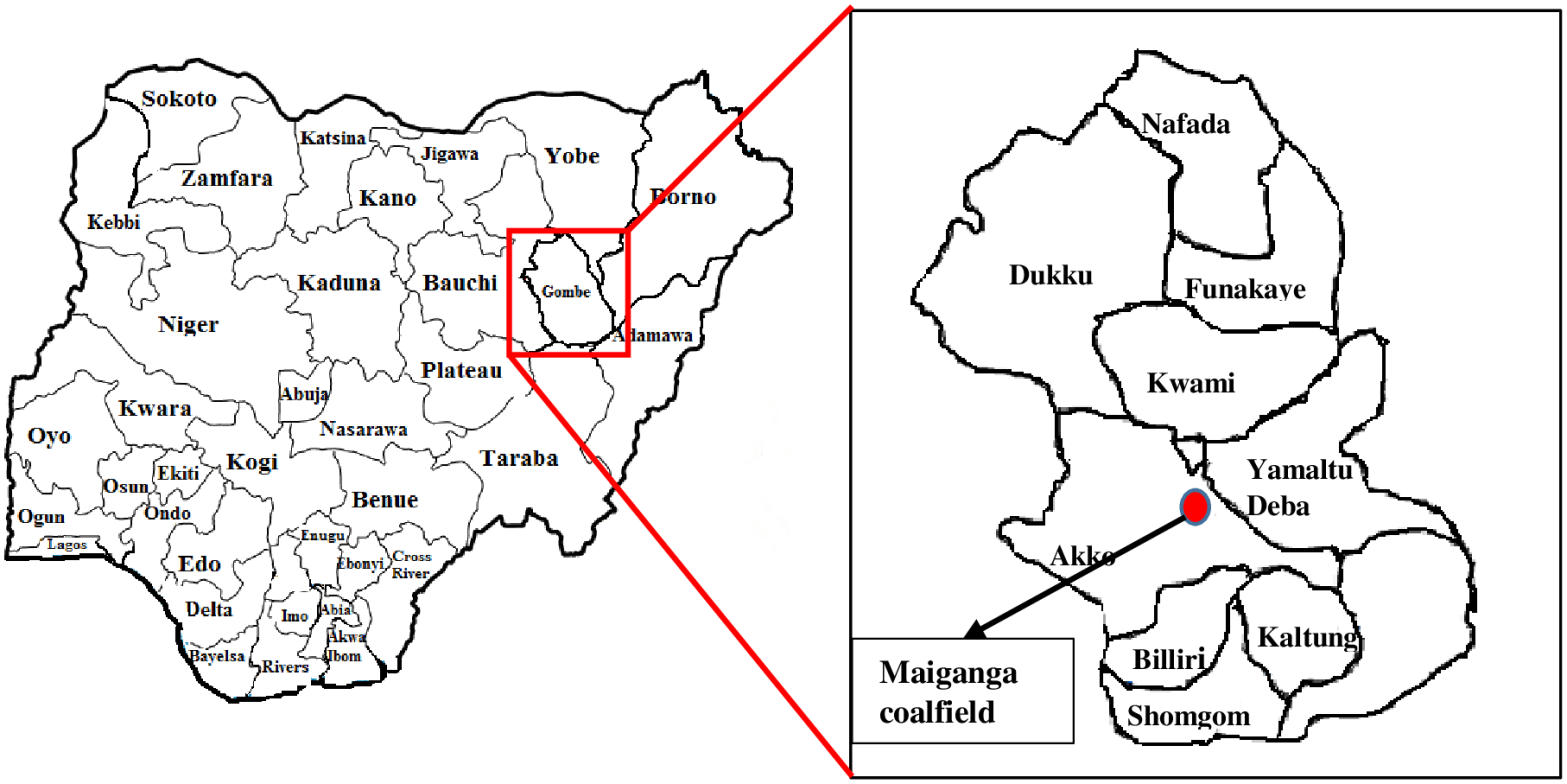
Map of Gombe State showing the project site.

Maiganga is a community located between latitude 10° 02′ to 10° 05° and longitude 11° 06′ to 11° 08′ in Akko local government area of Gombe, Northeast Nigeria (Fig 1). The Maiganga coal is hosted within the Maastrichtian Gombe formation located at the Northern Benue Trough of northeastern Nigeria. It is a low-rank subbituminous coal deposit that is targeted by Nigerian government for power generation. Exploration work is currently ongoing to ascertain the quantity and quality of this deposit. Presently, Maiganga coal is the main energy source for one of the leading cement production companies in Nigeria.

### Sample collection and processing

Thirty-three coal samples were collected from Maiganga coal mine for radiometric analysis. The samples were carefully collected from different points on the coal seam to satisfactorily represent the entire coal mine. The samples, each about 1.00 kg, were neatly packed in well labelled polyethylene bags, properly sealed, and transported by ship to Malaysia for analysis at the radiation laboratory, Physics Department, University of Malaya, Malaysia. The samples were air-dried at ambient temperature for 72 hours to attain constant weight in the laboratory. The dried samples were pulverized, sieved, and thoroughly homogenized. 375±1.0 g of the homogenized samples were carefully packed into well-labelled Marinelli beakers and properly sealed to prevent escape of radon. The sealed samples were stored for about five weeks to attain radiological (secular) equilibrium where the decay rates of the daughter nuclides and their respective parents become equal [5, 33, 34].

### Gamma spectrometric measurements

The gamma counting was done using a P-type Coaxial ORTEC, GEM-25 HPGe gamma ray detector with 57.5 mm crystal diameter and 51.5 mm thickness, shielded in a cylindrical lead shield with a fixed bottom in order to reduce the interference of background radiation from terrestrial and extra-terrestrial sources with the measured spectrum [35, 36]. The detector which has relative efficiency of 28.2% and 1.67 keV FWHM energy resolution at 1.33 MeV peak of ^60^Co, was coupled to ADCM data acquisition system with PCAII multi-channel analyser and set at operating voltage of +2800 V. Energy and efficiency calibrations were done using a cylindrical multi-nuclide gamma ray source with homogenously distributed activity in the same container geometry as the samples. The calibration source which was supplied by Isotopes Products Laboratories, (Valencia, CA 91355) has initial activity of 5.109μCi. The nuclides contained in the source along with their respective energies are: ^241^Am (59.541 keV), ^109^Cd (88.040 keV), ^57^Co (122.061 keV, 136.474 keV), ^203^Hg (279.195 keV), ^113^Sn (391.698 keV), ^85^Sr (514.007 keV), ^137^Cs (661.657 keV), ^88^Y (898.042 keV, 1836.063 keV), and ^60^Co (1173.22 keV, 1332.492 keV). The minimum detectable activity (MDA) at 95% confidence level for the detector was estimated following the equation [35]:

$$MDA\left(Bq/kg\right)=\frac{K_{\alpha }\sqrt{N_{B}}}{\eta \left(E\right)P_{\gamma }T_{c}M}$$ (1)

Where K_α_ is the statistical coverage factor equivalent to 1.645, N_B_ is the background count (cps), η (E) the photo-peak efficiency, P_γ_ is the probability of gamma emission, T_c_ the counting time(s), and M is the sample mass (kg). The characteristic gamma lines used to obtain the net activities of the respective nuclides are presented in Table 1. Using Eq (1) above, the MDA for the respective radionuclides of interest was calculated to be 0.60 Bq/kg for ^226^Ra, 0.70 Bq/kg for ^232^Th, and 2.40 Bq/kg for ^40^K.

**Table 1:** Decay data of radionuclides and the respective gamma lines used for activity determination.

Nuclides of interest	Detected nuclides	Halif-life	Decay mode (%)	γ-ray energy, E_γ_ (keV)	γ-ray intensity, I_γ_ (%)	Sources/origin
^226^Ra (^238^U)	^214^Pb	26.80 m	Β^−^ (100)	295.2228	18.42	^238^U (^226^Ra) series
				351.9321	35.60	^238^U (^226^Ra) series
	^214^Bi	19.90 m	α (0.02); β^−^ (99.98)	609.320	45.49	^238^U (^226^Ra) series
^228^Ra (^232^Th)	^228^Ac	6.15 h	α+β^−^ (100)	911.204	25.80	^232^Th series
				968.971	15.80	
	^208^Tl	3.053 m	Β^−^ (100)	583.187	85.00	^232^Th (^228^Ra) series
^40^K	^40^K	1.248E+09 y	EC (10.72); β^−^ (89.28)	1460.822	10.66	Primordial/terrestrial

Each sample and the background were counted for 86,400 seconds to achieve reasonable statistics at the radiation laboratory, Department of Physics, University of Malaya, Malaysia. The net count rate of the primordial radionuclides was obtained by subtracting the respective count rate from the background spectrum acquired for the same counting time [37, 38]. The specific activity concentrations of ^226^Ra, ^228^Ra, and ^40^K in all the samples investigated were calculated using the expression [35, 39, 40]:

$$A\left(Bq/kg\right)=\frac{CPS\times 1000}{\varepsilon_{\gamma }\times I_{\gamma }\times W}$$ (2)

where A (Bq/kg) is the specific activity, CPS is the net counts per second for each sample investigated, ε_γ_ (E) is the detector photo-peak efficiency at respective gamma-ray peak, I_γ_ is the corresponding gamma ray intensity, and W the mass of sample in g.

### Radiological hazard assessment

Based on the measured activity concentrations of ^226^Ra, ^232^Th, and ^40^K possible radiation health hazards to the exposed community were evaluated via the following hazard parameters:

#### Radium equivalent activity (Ra_eq_)

^226^Ra, ^232^Th, and ^40^K are not uniformly distributed in most environmental samples. A single parameter known as Radium equivalent activity (Ra_eq_) is defined with respect to radiation exposure which compares the activity of materials containing different elements of primordial radionuclides. Its definition also takes into account external and internal effective dose from radon and its decay progeny [27]. Ra_eq_ is measured in Bq kg^−1^ and defined based on the assumption that specific activity of 370 Bq kg^−1^ for ^226^Ra uniformly distributed in any environmental sample can result in annual effective dose of 1 mSv at 1 m above ground level [27, 41]. It is quantitatively expressed as [22]:

$$Ra_{eq}\left(Bq\;kg^{-1}\right)=A_{Ra}+1.43A_{Th}+0.077A_{K}$$ (3)

where A_Ra_, A_Th_, and A_K_ are the respective specific activities of ^226^Ra, ^232^Th, and ^40^K. The constants; 1, 1.43, and 0.077, represents the activity conversion rates for ^226^Ra, ^232^Th, and ^40^K in sequence, which result in same gamma dose rate at maximum permissible Ra_eq_ of 370 Bq kg^-1^.

#### External hazard index (H_ex_)

Radiation hazard incurred due to external exposure to gamma rays is quantified in terms of the external hazard index (H_ex_). The maximum permissible value for H_ex_ is unity, which corresponds to Ra_eq_ upper limit of 370 Bq kg^−1^ [27, 33]. H_ex_ is calculated from the equation:

$$H_{ex}=\frac{Ra_{eq}}{370}$$ (4)

Or:

$$H_{ex}=\frac{A_{Ra}}{370}+\frac{A_{Th}}{259}+\frac{A_{K}}{4810}$$ (5)

where A_Ra_, A_Th_, and A_K_ are the specific activities of ^226^Ra, ^232^Th, and ^40^K, respectively. It is assumed that 370 Bq kg^−1^ of ^226^Ra, 259 Bq kg^−1^ of ^232^Th, and 4810 Bq kg^−1 40^K, produce the same gamma dose rate [2, 4, 10]

#### Internal hazard index (H_in_)

Respiratory organs are in danger of radiation exposure to radon and its carcinogenic daughters. The internal radiation exposure is quantified by the internal hazard index (H_in_) given by UNSCEAR [22]:

$$H_{in}=\frac{A_{Ra}}{185}+\frac{A_{Th}}{259}+\frac{A_{K}}{4810}$$ (6)

where, A_Ra_, A_Th_, and A_K_ are the specific activities of ^226^Ra, ^232^Th, and ^40^K, respectively. UNSCEAR [22] provided that the value of the above indexes must be less than unity for the radiation hazard to be regarded as insignificant.

#### Representative gamma index (I_γr_)

The representative gamma index (I_γr_) is a screening parameter for materials of possible radiation health challenge [42]. It is calculated using the equation [43–45]:

$$I_{\gamma r}=\frac{A_{Ra}}{150}+\frac{A_{Th}}{100}+\frac{A_{K}}{1500}$$ (7)

where A_Ra_, A_Th_, and A_K_ are the specific activity concentrations of ^226^Ra, ^232^Th, and ^40^K, respectively, in Bq kg^−1^. The numerical denominators of 150, 100, and 1500, are specific exposure rates for ^226^Ra, ^232^Th, and ^40^K, respectively that yield a sum of I_γr_ ≤ 1, which corresponds to an annual effective dose of ≤ 1mSv, to satisfy the dose criteria [46, 47].

#### Excess lifetime cancer risk (ELCR)

Excess life-time cancer risk (ELCR) was estimated from annual effective dose equivalent using the equation [45, 47]:

ELCR=AEDE×DL×RF (8)

where AEDE, DL, and RF are the annual effective dose equivalent, duration of life (70 years), and risk factor (0.05Sv^-1^), respectively. Ravisankar et al [45], defined the risk factor as fatal cancer risk per Sievert, which according to Taskin et al [47], is assigned a value of 0.05 by ICRP 60 for the public for stochastic effects.

### Occupational risk estimation

Open-cast mining is a surface mining technique employed for coal mining in Maiganga coalfield. This technique involves the removal of large volumes of overburden waste rock (mine tailings) which consist of relatively loose, non-compacted debris that are dumped indiscriminately on the surface within the vicinity of the mine. As a result, workers are continually exposed to enhanced radiation dose through three primary exposure pathways:

1. External exposure to gamma radiation from mined coal and the exposed tailings,
2. Internal exposure from inhalation of coal dust and contaminated air,
3. Internal exposure from any accidental ingestion of coal.

Radiation doses received by exposed individual/group via the exposure routes are calculated from the specific activities of radionuclides measured in the samples by applying relevant dose conversion coefficients provided by the International Commission on Radiological Protection (ICRP). These doses, when added together, result in the total effective dose delivered by the radionuclides. The risk of any adverse radiation induced health hazard is dependent on the total effective dose.

Dose from external exposure to gamma radiation is estimated from the equation [48, 49]:

$${\text{D}}_{\text{ext}}=\sum_{\text{i}}{\text{A}}_{\text{i}}{\text{ C}}_{\text{ext},\text{ i}}{\text{ T}}_{\text{e}}$$ (9)

where A_i_ is the specific activity of nuclide i in Bq kg^-1^, C_ext, i_ is the effective dose coefficient for nuclide i in the contaminated surface measured in Sv h^-1^/Bq g^-1^, and T_e_ is the exposure duration in number of years.

Internal exposure from inhalation of coal dust is calculated using the relation [48]:

$${\text{D}}_{\text{inh}}=\sum_{\text{i}}{\text{A}}_{\text{i}}{\text{ C}}_{\text{inh},\text{i}}{\text{ η}}_{\text{inh}}{\text{ D}}_{\text{f}}{\text{ T}}_{\text{e}}$$ (10)

where C_inh,_ is the dose coefficient for inhalation of nuclide i measured in Sv Bq^-1^, η_inh_ is the breathing rate measured in m^3^ h^-1^, and D_f_ is the dust loading factor. T_e_ and A_i_ are as defined in Eq (9).

Internal dose from accidental ingestion of radionuclides is estimated from the equation

$${\text{D}}_{\text{ing}}=\sum_{\text{i}}{\text{A}}_{\text{i}}{\text{ C}}_{\text{ing},\text{i }}\eta_{\text{ing }}{\text{T}}_{\text{e}}$$ (11)

where C_ing,_ is the dose coefficient for ingestion of nuclide i, measured in Sv Bq^-1^; η_ing_ is the ingestion rate for adults, measured in kg h^-1^; and A_i_ and T_e_ remained as defined in Eq (9).

## Results and Discussion

Descriptive statistics of activity concentrations in Bq kg^−1^ of ^226^Ra, ^232^Th, and ^40^K with their respective uncertainty levels of ±σ, involving the minimum, maximum, mean, and standard deviation of Maiganga coal samples are presented in Table 2. The range of activities obtained for the studied coal showed very low concentrations for ^226^Ra (from 3.73±0.1 to 16.26±0.3 Bq kg^−1^), ^232^Th (between 2.02±0.1 and 11.29±0.3 Bq kg^−1^), and for ^40^K (from 6.69±0.1 to 44.08±0.7 Bq kg^−1^), with respective average values of 8.18±0.3, 6.97±0.3, and 27.38±0.8 Bq kg^−1^.

**Table 2:** Activity concentrations and radiation hazard indices of coal from Maiganga coalfield.

Sample ID	Activity concentrations (Bq kg-1)				Hazard indexes (≤ 1)			
	226Ra	232Th	40K	Raeq	Hex	Hin	Iγr	ELCR (x10-3)
**MCS 01**	12.66±0.6	11.04±0.6	17.92±1.0	29.83	0.08	0.11	0.21	0.06
**MCS 02**	12.82±0.7	10.55±0.6	19.09±1.0	29.38	0.08	0.11	0.20	0.06
**MCS 03**	6.99±0.4	3.54±0.3	7.71±1.0	12.65	0.03	0.05	0.09	0.02
**MCS 04**	10.79±0.6	9.14±0.5	13.33±1.0	24.88	0.07	0.10	0.17	0.05
**MCS 05**	6.62±0.4	3.35±0.3	9.18±1.0	12.11	0.03	0.05	0.08	0.02
**MCS 06**	10.83±0.6	8.33±0.5	12.89±1.0	23.73	0.06	0.09	0.16	0.04
**MCS 07**	11.43±0.6	9.29±0.5	15.11±1.0	25.88	0.07	0.10	0.18	0.05
**MCS 08**	10.51±0.6	9.06±0.5	12.06±1.0	24.40	0.07	0.09	0.17	0.05
**MCS 09**	13.09±0.3	8.07±0.2	36.63±0.6	27.46	0.07	0.11	0.19	0.05
**MCS 10**	12.99±0.2	8.10±0.2	34.43±0.5	27.22	0.07	0.11	0.19	0.05
**MCS 11**	9.36±0.2	8.33±0.2	29.85±0.5	23.56	0.06	0.09	0.17	0.04
**MCS 12**	8.29±0.5	7.24±0.4	16.17±0.9	19.89	0.05	0.08	0.14	0.04
**MCS 13**	7.39±0.4	6.13±0.4	14.98±0.8	17.30	0.05	0.07	0.12	0.03
**MCS 14**	16.26±0.3	11.29±0.3	37.55±0.6	35.30	0.10	0.14	0.25	0.07
**MCS 15**	15.95±0.3	11.26±0.3	38.56±0.6	35.01	0.09	0.14	0.24	0.07
**MCS 16**	6.18±0.4	7.07±0.4	15.51±0.9	17.48	0.05	0.06	0.12	0.03
**MCS 17**	8.55±0.2	9.36±0.2	6.69±0.1	22.46	0.06	0.08	0.16	0.04
**MCS 18**	8.79±0.2	7.44±0.2	34.34±0.6	22.08	0.06	0.08	0.16	0.04
**MCS 19**	7.61±0.2	5.83±0.2	41.32±0.7	19.12	0.05	0.07	0.14	0.04
**MCS 20**	7.97±0.1	7.34±0.1	44.08±0.7	21.86	0.06	0.08	0.16	0.04
**MCS 21**	5.05±0.1	6.87±0.1	36.11±0.6	17.65	0.05	0.06	0.13	0.03
**MCS 22**	4.48±0.1	4.04±0.1	31.92±0.5	12.71	0.03	0.05	0.09	0.02
**MCS 23**	3.73±0.1	2.02±0.1	29.92±0.5	8.92	0.02	0.03	0.06	0.02
**MCS 24**	6.01±0.1	7.60±0.2	35.73±0.6	19.62	0.05	0.07	0.14	0.04
**MCS 25**	5.81±0.1	7.33±0.2	38.49±0.6	19.26	0.05	0.07	0.14	0.04
**MCS 26**	5.35±0.1	5.92±0.1	35.29±0.6	16.52	0.04	0.06	0.12	0.03
**MCS 27**	4.80±0.1	5.71±0.1	36.35±0.6	15.75	0.04	0.06	0.11	0.03
**MCS 28**	4.40±0.1	4.38±0.1	38.89±0.6	13.66	0.04	0.05	0.10	0.03
**MCS 29**	5.37±0.1	5.13±0.2	39.18±0.7	15.73	0.04	0.06	0.11	0.03
**MCS 30**	4.70±0.3	4.10±0.3	31.35±1.6	12.97	0.04	0.05	0.09	0.03
**MCS 31**	5.09±0.3	4.92±0.4	30.97±1.6	14.52	0.04	0.05	0.10	0.03
**MCS 32**	4.90±0.3	4.89±0.3	30.27±1.6	14.22	0.04	0.05	0.10	0.03
**MCS 33**	5.22±0.3	5.35±0.4	31.62±1.7	15.31	0.04	0.06	0.11	0.03
**Min**	**3.73±0.1**	**2.02±0.1**	**6.69±0.1**	**8.92**	**0.02**	**0.03**	**0.06**	**0.02**
**Max**	**16.26±0.3**	**11.29±0.3**	**44.08±0.7**	**35.30**	**0.10**	**0.14**	**0.25**	**0.07**
**Mean**	**8.18±0.3**	**6.97±0.3**	**27.38±0.8**	**20.26**	**0.05**	**0.08**	**0.14**	**0.04**

**Fig 2:**
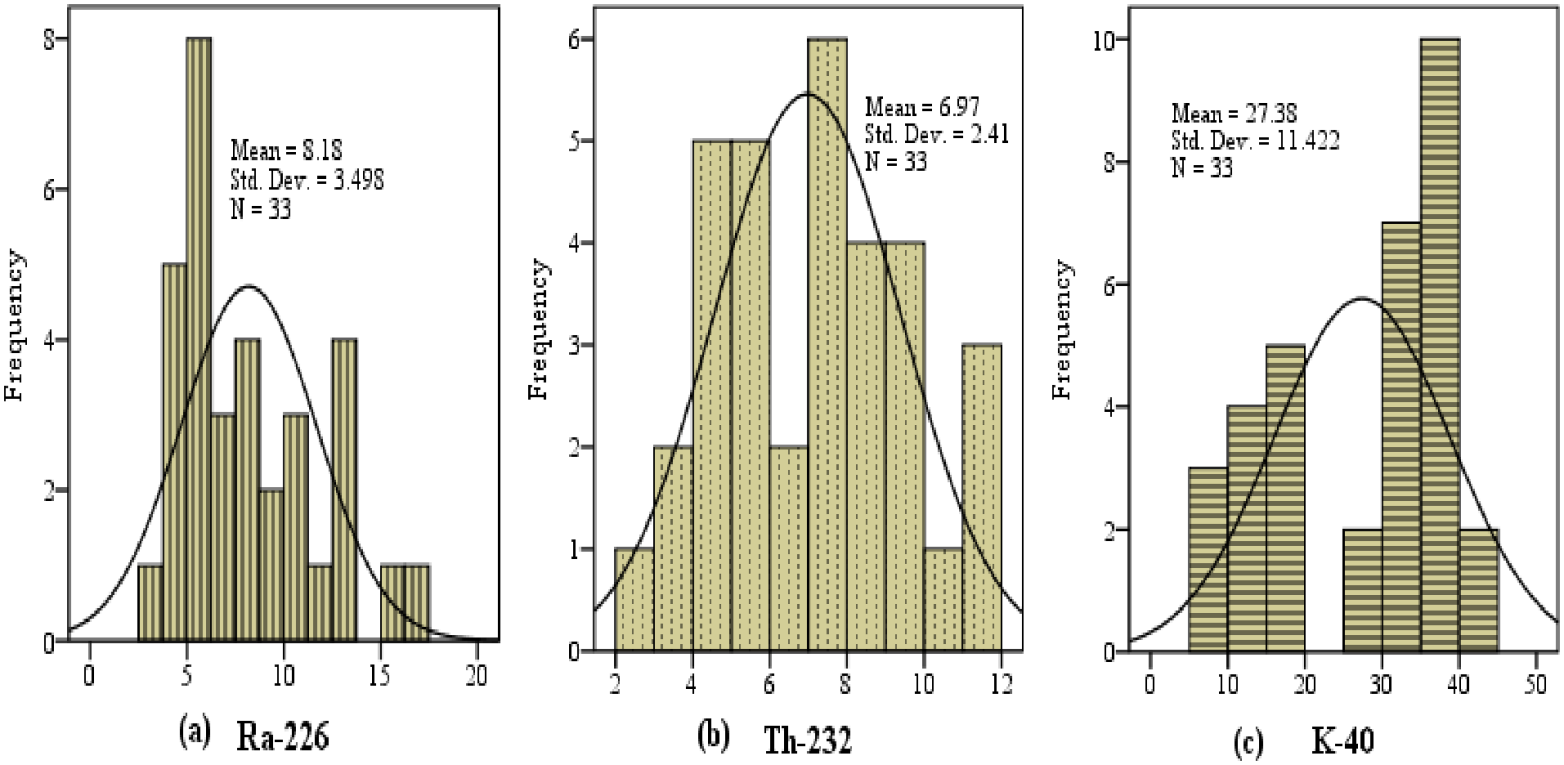
Frequency distribution histograms of (a) ^226^Ra, (b) ^232^Th and (c) ^40^K in Maiganga coal.

The normal (Bell-shaped) frequency distribution histograms shown in Fig 2 demonstrated the even distribution of primordial radionuclides in Maiganga coal.

Uranium is present mainly in the carbonaceous components of sedimentary rocks and accumulates in both mineral and organic fractions of coal during coalification, while thorium is contained in common phosphate minerals and accumulates in inorganic phases [1, 8, 10]. The mean activity concentrations of ^226^Ra, ^232^Th, and ^40^K recorded for the studied coal were found to be lower than those obtained for similar studies around the world (Table 3). The values were also below the world average values for coal provided by UNSCEAR [50] as seen in Table 3, showing that the mean concentrations of natural radionuclides in coal are generally lower than their respective mean values in the earth crust [22].

**Table 3:** Comparison of Activity Concentrations (Bq kg^−1^) of ^226^Ra, ^232^Th and ^40^K of Maiganga Coal with the world average values and those of other published works.

Country	^226^Ra	^232^Th	^40^K	References
Hong Kong, China	17	20	24	Tso and Leung [30]
Kolaghat, India	25.0–49.9	39.3–55.2	120.8–151	Mandal and Sengupta [1]
Baoji, China	26.3	36.6	99.8	Lu et al. [2]
Cayrrhan, Turkey	14.55	11.12	123.01	Cevik et al. [5]
Spain	64	18	104	Mora et al. [31]
Greece	133	18	108	Papastefanou [17]
Serbia	16	12	60	Kisic et al. [32]
Kosovo	9	9	36	Hasani et al. [13]
Nigeria (Northeast)	8.18	6.97	27.38	Present study
World average	20	20	50	UNSCEAR [50]

Coal is an indispensable fuel for power generation and a base industrial raw material in many developed and developing countries including Nigeria. It is therefore important that the radiological health effects associated with its exploitation and utilization be assessed.

Radiation hazard indices which include the radium equivalent activity (Ra_eq_), external hazard index (H_ex_), internal hazard index (H_in_), gamma index (I_γr_), and excess life cancer risk (ELCR) were computed from Eqs (3) and (8) and the results presented in columns 4 to 7 of Table 2. The calculated values for Ra_eq_ activity index for Maiganga coal were in the range of 8.92 to 35.30 Bq kg^−1^ with a mean value of 20.26 Bq kg^−1^ and standard deviation of 6.0 Bq kg^−1^. The corresponding external hazard index recorded an average value of 0.05 for the studied coal. These values were found to be lower than the respective maximum values of 370 Bq kg^−1^ and one, recommended by UNSCEAR [22]. The internal hazard index, H_in_, which describes the degree of internal exposure by radon and its decay products, recorded an average value of 0.08, while mean gamma index, I_γr_, for the Maiganga coal was 0.14 (Table 2). These values were less than the safety limit of unity stipulated by UNSCEAR [22], indicating the non-hazardous nature of Maiganga coal. The excess lifetime cancer risk (ELCR) calculated for Maiganga coal varied between 0.02 x 10^−3^ and 0.07×10^−3^ with a mean value of 0.04 ×10^−3^ and standard deviation of 0.01×10^−3^. This value was found to be less than the precautionary limits of 0.29×10^−3^ set by UNSCEAR [22] and 0.05 prescribed by ICRP for low-level radiations. Generally, the estimated radiation hazard indices presented in Table 2 for Maiganga coal were found to be lower than their respective safety limits prescribed by UNSCEAR. Thus, the exploitation and utilization of Maiganga coal either for power generation or other industrial and domestic uses does not pose any significant radiological impact to the coal workers, the coal users, and the general environment.

### Correlation coefficients

The interdependency and natural relationships existing among the measured radiological parameters for Maiganga coal were evaluated by Pearson’s correlation matrix with the alpha testing level at P<0.05 for coal samples (n = 33) using the statistical program for social science (SPSS 22.0). The calculated correlation coefficients are presented in Table 4. Very strong positive relationship was found to exist between ^226^Ra, and ^232^Th (r = +0.85), while weak negative degree of association existed between ^40^K and ^232^Th (r = -0.12), and ^226^Ra (r = -0.15). The strong positive correlation between ^226^Ra and ^232^Th may not be unconnected with the fact that radium and thorium decay series have a common origin and exist together in nature [48]. Furthermore, all the estimated radioactive variables were strongly correlated with one another positively, and also with ^226^Ra, and ^232^Th (r ≥ +0.91). ^40^K, on the other hand, exhibited very weak relationship with all the radiological variables (-0.02 ≤ r ≤ +0.05). This indicated that the emission of gamma radiation is principally due to ^226^Ra, and ^232^Th contents of Maiganga coal.

**Table 4:** Correlation matrix of radiological variables for Maiganga coal.

**Variables**	**^226^Ra**	**^232^th**	**^40^K**	**Ra_eq_**	**H_ex_**	**H_in_**	**I_γr_**	**ELCR**
^**226**^**Ra**	1.00							
^**232**^**Th**	0.85	1.00						
^**40**^**K**	-0.15	-0.12	1.00					
**Ra**_**eq**_	0.95	0.96	-0.01	1.00				
**H**_**ex**_	0.93	0.96	-0.02	0.99	1.00			
**H**_**in**_	0.97	0.92	-0.02	0.99	0.97	1.00		
**I**_**γr**_	0.94	0.96	0.03	1.00	0.98	0.98	1.00	
**ELCR**	0.91	0.93	0.05	0.97	0.97	0.96	0.97	1.00

### Total effective dose from coal mining operation

Eqs (9), (10) and (11) were used to calculate the radiation doses incurred by coal workers from exposure to ^226^Ra, ^232^Th, and ^40^K in Maiganga coal through the three exposure pathways described earlier. Relevant dose conversion coefficients and other parameters adopted for the calculations are presented in Table 5. The results of the calculated radiation doses and the total annual effective dose are presented in Table 6.

**Table 5:** Dose coefficients and other risk parameters adopted in this study.

Parameters	Values				Reference
**Breathing rate, η**_**inh**_ **(m**^**3**^ **h**^**-1**^**)**	1.69				Mustapha et al. [47]
**Dust loading factor, D**_**f**_ **(g m**^**-3**^**)**	1×10^−3^				Degrand & Lepicard [51]
**Ingestion rate, η**_**ing**_ **(kg h**^**-1**^**)**	5×10^−6^				Mustapha et al. [47]
**Duration of exposure, T (h y**^**-1**^**)**	2000				
		^**226**^**Ra**	^**232**^**Th**	^**40**^**K**	
**Effective dose coefficient, C**_**ext**_ **(nSv h**^**-1**^**/Bq kg**^**-1**^**)**		9.929	0.003	1.175	Mustapha et al. [47]
**Dose coefficient for inhalation, C**_**inh**_ **(Sv Bq**^**-1**^**)**		2.2E-06 (m)	2.9E-05 (m)	3.0E-09 (f)	ICRP 119 [52]
**Dose coefficient for ingestion, C**_**ing**_ **(Sv Bq**^**-1**^**)**		2.8E-07 (m)	2.2E-07 (m)	6.2E-09 (f)	ICRP 119 [52]

m, f, denotes moderate, and fast rate of absorption from respiratory tract respectively

**Table 6:** Calculated effective dose for workers in Maiganga coalfield from the three exposure routes.

Sample ID	Effective dose (μSv y^-1^)			Total effective dose
	D_ext_	D_inh_	D_ing_	(mSv y^-1^)
**MCS 01**	0.29	11.76	0.03	1.21 × 10^−2^
**MCS 02**	0.30	11.29	0.03	1.16 × 10^−2^
**MCS 03**	0.16	3.99	0.01	4.17 × 10^−3^
**MCS 04**	0.25	9.76	0.02	1.00 × 10^−2^
**MCS 05**	0.15	3.77	0.01	3.94 × 10^−3^
**MCS 06**	0.25	8.97	0.02	9.23 × 10^−3^
**MCS 07**	0.26	9.96	0.02	1.02 × 10^−2^
**MCS 08**	0.24	9.67	0.02	9.92 × 10^−3^
**MCS 09**	0.35	8.89	0.03	9.26 × 10^−3^
**MCS 10**	0.34	8.91	0.03	9.28 × 10^−3^
**MCS 11**	0.26	8.86	0.02	9.13 × 10^−3^
**MCS 12**	0.20	7.71	0.02	7.93 × 10^−3^
**MCS 13**	0.18	6.56	0.01	6.75 × 10^−3^
**MCS 14**	0.41	12.28	0.03	1.27 × 10^−2^
**MCS 15**	0.41	12.22	0.03	1.27 × 10^−2^
**MCS 16**	0.16	7.39	0.01	7.56 × 10^−3^
**MCS 17**	0.19	9.81	0.02	1.00 × 10^−2^
**MCS 18**	0.26	7.95	0.02	8.22 × 10^−3^
**MCS 19**	0.25	6.28	0.02	6.54 × 10^−3^
**MCS 20**	0.26	7.79	0.02	8.07 × 10^−3^
**MCS 21**	0.19	7.11	0.01	7.31 × 10^−3^
**MCS 22**	0.16	4.30	0.01	4.47 × 10^−3^
**MCS 23**	0.14	2.26	0.01	2.41 × 10^−3^
**MCS 24**	0.20	7.89	0.01	8.11 × 10^−3^
**MCS 25**	0.21	7.62	0.01	7.84 × 10^−3^
**MCS 26**	0.19	6.20	0.01	6.40 × 10^−3^
**MCS 27**	0.18	5.95	0.01	6.14 × 10^−3^
**MCS 28**	0.18	4.62	0.01	4.81 × 10^−3^
**MCS 29**	0.20	5.44	0.01	5.64 × 10^−3^
**MCS 30**	0.17	4.37	0.01	4.54 × 10^−3^
**MCS 31**	0.17	5.20	0.01	5.39 × 10^−3^
**MCS 32**	0.17	5.16	0.01	5.33 × 10^−3^
**MCS 33**	0.18	5.64	0.01	5.83 × 10^−3^
**Min**	0.14	2.26	0.01	2.41 × 10^−3^
**Max**	0.41	12.28	0.03	1.27 × 10^−2^
**Mean**	0.23	7.44	0.02	7.69 × 10^−3^

The calculated effective dose due to external exposure to contaminated surfaces varied between 0.14 and 0.41 μSv y^-1^. The effective dose delivered to the workers through the inhalation pathway ranged from 2.26 to 12.28 μSv y^-1^, while that from accidental ingestion of radionuclides recorded a mean value of 0.02 μSv y^-1^. Total annual effective dose received through the three exposure routes varied from 2.41 × 10^−3^ to 1.27 × 10^−2^ mSv y^-1^, with an average value of 7.69 × 10^−3^ mSv y^-1^. The results showed that the most significant occupational exposure pathway is the inhalation of coal dust which accounted for about 97% of the total average annual effective dose to workers. The average total annual effective dose obtained in this study is below the precautionary limit of 1.0 mSv y^-1^ set by the International Atomic Energy Agency, IAEA [53], indicating that the exploitation of coal from Maiganga coalfield does not constitute any deleterious radiological threat to coal workers. Workers welfare should however, not be taken for granted.

## Conclusions

Thirty-three coal samples from Maiganga coal mine Gombe Northeast Nigeria were characterized for their ^226^Ra, ^232^Th, and ^40^K activity concentrations using HPGe gamma spectrometer. The mean activities of ^226^Ra, ^232^Th, and ^40^K were 8.18±0.3, 6.97±0.3, and 27.38±0.8 Bq kg^−1^ respectively. These values were lower than the world average values of 20, 20, and 50 Bq kg^−1^, respectively, for coals provided by UNSCEAR. The values were also below those obtained for similar studies around the world. The calculated average value for Ra_eq_, was 20.26 Bq kg^−1^. Similarly, H_ex_, H_in_, and I_γr_ recorded average values of 0.05, 0.08, and 0.14, respectively for the studied coal samples. These values were below their respective precautionary limits set by UNSCEAR. Furthermore, mean total annual effective dose of 7.69 x 10^−3^ mSv y^-1^ received by coal workers was found to be below safety criterion of 1.0 mSv y^-1^ set by ICRP. Although inhalation of coal dust was identified as the most significant exposure pathway for workers, the overall results showed that Maiganga coal is radiologically safe for exploitation and utilization either as fuel for power generation, as industrial raw material or for domestic services.
